# Prevalence and mechanisms of fluoroquinolone-resistant *Escherichia coli* among sheltered companion animals

**DOI:** 10.1099/acmi.0.000077

**Published:** 2019-11-05

**Authors:** Kaoru Umeda, Atsushi Hase, Akira Fukuda, Masashi Matsuo, Tomoaki Horimoto, Jun Ogasawara

**Affiliations:** ^1^​ Division of Microbiology, Osaka Institute of Public Health, 8-34, Tojo-cho, Tennoji-ku, Osaka 543-0026, Japan; ^2^​ Osaka Municipal Animal Care and Control Center, 2-5-74, Shibatani, Suminoe-ku, Osaka 559-0021, Japan

**Keywords:** fluoroquinolone-resistant *Escherichia coli*, companion animals, QRDR, PMQR

## Abstract

To better understand the prevalence of fluoroquinolone-resistant *Escherichia coli* among sheltered companion animals, we conducted a screening study of 38 dogs and 78 cats and investigated the resistance mechanisms and characteristics of the isolates. Fluoroquinolone-resistant *E. coli* was detected in 18 dogs (47.4 %) and 14 cats (17.9 %). The isolates carried one to four mutations in the *gyr*A, *par*C and *par*E genes of the quinolone resistance-determining region, and the number of mutations was proportional to the MIC for ciprofloxacin. For plasmid-mediated quinolone resistance, *aac-(6′)-Ib-cr* was detected in nine isolates, *qnr*S in five isolates and *qnr*B in one isolate. A relationship between the presence of these genes and MIC for ciprofloxacin was not apparent. Statistical analysis indicated that fluoroquinolone-resistant *E. coli* was widely distributed among sheltered companion animals with various attributes. This may relate to the wide dissemination of fluoroquinolone resistance among humans and other animals in Japan.

## Data Summary

This paper did not require other supporting external data.

**Impact Statement**

Fluoroquinolones are broad-spectrum antimicrobials that are useful for the treatment of bacterial infections. However, fluoroquinolone resistance has increased in the clinical field. This paper first clarified that fluoroquinolone-resistant *Escherichia coli* was widely distributed among sheltered companion animals with a wide range of individual attributes. This finding is important with regard to the circulation of fluoroquinolone resistance among humans, among animals and in the environment. It may have considerable influence on the treatment of infectious disease in both animals and humans given that 46.9 % of isolates were classified as multidrug-resistant bacteria. In order to lower the overall level of fluoroquinolone resistance, it will be necessary to reduce fluoroquinolone use and continue surveillance not only in the human clinical field but also in the veterinary field.

## Introduction

Fluoroquinolones are broad-spectrum and effective bactericidal antimicrobials that have been approved for use in the treatment of various bacterial infections. Recently, fluoroquinolone resistance has increased in the clinical field nationwide [1]. The Japan Nosocomial Infections Surveillance (JANIS) reported that 60.2 and 59.0 % of *Escherichia coli* isolates from inpatient specimens obtained in 2015 and 2016, respectively, were resistant to levofloxacin [2]. The main fluoroquinolone resistance mechanism is the chromosomal mutation of quinolone resistance-determining region (QRDR) in DNA gyrase and topoisomerase IV. Another mechanism, plasmid-mediated quinolone resistance (PMQR), was also found to contribute to quinolone resistance [3–5].

We have previously reported the prevalence of cephalosporin-resistant *Enterobacteriaceae* among sheltered companion animals; 14.6 % of dogs and 11.0 % of cats harboured cephalosporin-resistant bacteria, and 33 % of these isolates were also resistant to fluoroquinolone. Based on the distribution of several types of antimicrobial-resistant bacteria among animals with a wide range of individual attributes, we concluded that companion animals may play a bridging role in the circulation of antimicrobial-resistant bacteria from humans and other origins [6]. Therefore, surveillance of sheltered companion animals may be useful in determining the prevalence of antimicrobial-resistant bacteria.

Several studies have demonstrated the presence and characteristics of fluoroquinolone-resistant *E. coli* among companion animals [7–11]. However, most of them included results from clinical isolates obtained from animal hospitals and did not aim to demonstrate the prevalence of fluoroquinolone-resistant bacteria among a wide range of companion animals.

Based on the above background, we first established a means to understand the prevalence of fluoroquinolone-resistant *E. coli* among diverse dogs and cats, and then screened a number of sheltered animals and investigated the resistance mechanisms and genetic characteristics of the isolates.

## Methods

Faecal samples or rectal swabs were collected between April 2016 and January 2017 from 38 dogs and 78 cats brought to Osaka Municipal Animal Care and Control Center. The sampling and classifying of animals by individual attributes were done as in our previous report [6]. Samples were inoculated on MacConkey agar (Nissui Pharmaceutical), supplemented with ciprofloxacin at 4 mg l^−1^, and incubated at 37 °C for 20 h. Subculturing was performed on up to five *E. coli *-like colonies. Bacterial species were identified by biochemical tests with an API 20E identification kit (bioMérieux). *E. coli* O-serotyping, *E. coli* phylogenetic grouping and PFGE typing were carried out as in our previous report [6]. MICs were determined via a Dry Plate EIKEN (Eiken Chemical) and ETEST (bioMérieux) according to the manufacturers' instructions. The results were interpreted according to the Clinical and Laboratory Standards Institute (CLSI) guidelines [12]. Mutations in QRDR genes were confirmed by PCR amplification and sequencing [13]. PMQR genes were screened by PCR [14–16]. The β-lactamase-producing phenotype and β-lactamase genes were detected as in our previous report [6]. When several isolates were cultured from the same animal, they were designated as the same clone if they exhibited the same PFGE patterns. The correlation between the prevalence of fluoroquinolone-resistant *E. coli* and individual attributes was estimated using Fisher’s exact test. A *P* value of <0.05 was considered significant.

## Results

The correlation between individual animal attributes (sex, age group, presence/absence of an owner, health status and enrofloxacin medication treatment) and the prevalence of fluoroquinolone-resistant *E. coli* is presented in Table 1. Fluoroquinolone-resistant *E. coli* was detected in 18 out of 38 dogs (47.4 %) and 14 out of 78 cats (17.9 %). Statistical analysis showed that prevalence among dogs was significantly higher than among cats (*P*=0.0017), and prevalence in males was significantly higher than in females among both dogs (*P*=0.0064) and cats (*P*=0.0088). No significant correlations were found relating to the presence/absence of an owner or treatment with enrofloxacin medication in the animal shelter.

**Table 1.** Correlation between animal attributes and prevalence of fluoroquinolone-resistant *E. coli *.

Animal attributes		Detected/population (%)	
		Dog*^a^* 18/38 (47.4 %)	Cat*^a^* 14/78 (17.9 %)
Sex	Male	16/25 (64.0 %)^*b*^	11/36 (30.6 %)^*c*^
	Female	2/13 (15.4 %)^*b*^	3/42 (7.1 %)^*c*^
Age group	Young	0/4 (0 %)	5/20 (25.0 %)
	Mature to old	18/34 (52.9 %)	9/58 (15.5 %)
Owner^*d*^	Presence	13/24 (54.2 %)	1/16 (6.3 %)
	Absence	5/14 (35.7 %)	13/62 (21.0 %)
Health status^*e*^	Healthy	15/32 (46.9 %)	11/59 (18.6 %)
	Unhealthy	3/6 (50.0 %)	3/19 (15.8 %)
Enrofloxacin^*f*^	Medication	2/5 (40.0 %)	0/0 (0 %)
	No medication	16/33 (48.5 %)	14/78 (17.9 %)

*a,* A significant difference (*P*=0.0017) was detected.

*b,* A significant difference (*P*=0.0064) was detected.

*c,* A significant difference (*P*=0.0088) was detected.

*d,* Presence or absence of owner when admitted to an animal shelter.

*e,* Unhealthy group included animals with emaciation, weakness, injury, symptoms of illness, or malnutrition.

*f,* Enrofloxacin medication for treatment in an animal shelter.

The resistance mechanisms and characteristics of 32 fluoroquinolone-resistant *E. coli* isolates are given in Table 2. All isolates showed MICs ≥256 µg ml^−1^ for nalidixic acid and demonstrated at least one mutation in the QRDR. Four isolates with MICs of 2–3 µg ml^−1^ for ciprofloxacin harboured a single mutation in *gyr*A. Fourteen isolates with MICs of 3–24 µg ml^−1^ for ciprofloxacin carried two or three mutations in the QRDR: the combination of a double mutation (except for isolate 37E-1) in *gyr*A and a single mutation in *par*C. Fourteen isolates with MICs ≥32 µg ml^−1^ for ciprofloxacin carried a total of four mutations in the QRDR: the combination of a double mutation in *gyr*A, a single mutation in *par*C and a single mutation in *par*E; or the combination of a double mutation in *gyr*A and a double mutation in *par*C. For PMQR, nine isolates harboured *aac-(6′)-Ib-cr*, five isolates harboured *qnr*S and one isolate harboured *qnr*B. The *qnr*S gene was always harboured simultaneously with *aac-(6′)-Ib-cr*. A relationship between the presence of PMQR and ciprofloxacin MIC values was not apparent. There were seven isolates resistant to cefotaxime and six isolates produced β-lactamase from the various β-lactamase gene(s). Extended-spectrum β-lactamase (ESBL) genes (CTX-M-14 or CTX-M-27) were harboured simultaneously with *aac-(6′)-Ib-cr*, while the DHA-1 gene was harboured with *qnrB*. For the O-serotype, most of the *E. coli* isolates were untyped, except for serotypes O1, O25 and O157, each from one isolate. For phylogenetic grouping, three isolates belonged to phylogenetic group B2, and 11 isolates belonged to group D, while the remaining isolates belonged either to group A or to group B1.

**Table 2.** Characteristics of fluoroquinolone-resistant *E. coli* isolates from dogs and cats

Isolate ID	Animal species	MIC(μg ml^−1^)^*a*^ for		QRDR*^b^*					PMQR^*c*^	O-serotype	Phylogenetic group	MIC (μg ml^−1^)^*a*^ for	β-Lactamase (β-lactamase gene)	PFGE type^*d*^
		Ciprofloxacin	Nalidixic acid	*gyr*A		*par*C		*par*E				Cefotaxime		
				Ser83	Asp87	Ser80	Glu84	Ser458						
51–1	Dog	2	>256	Leu83	－	－	－	－	*qnr*S, *aac-(6′)-Ib-cr*	UT	D	<1		9′
64–2	Dog	2	256	Leu83	－	－	－	－	*qnrS, aac-(6′)-Ib-cr*	UT	D	<1		9′′′
72–1	Cat	2	>256	Leu83	－	－	－	－	*qnrS, aac-(6′)-Ib-cr*	UT	D	<1		9′′′′
63–1	Dog	3	>256	Leu83	－	－	－	－	*qnrS, aac-(6′)-Ib-cr*	UT	D	<1		9′′
32–1	Dog	3	>256	Leu83	Asn87	Ile80	－	－		UT	A	4	AmpC (CMY-2)	6
26–1	Cat	4	>256	Leu83	Tyr87	Ile80	－	－		O157^*e*^	B1	<1		10
89–1	Cat	4	>256	Leu83	Asn87	Ile80	－	－		UT	B1	<1		13
37E-1	Cat	6	>256	Leu83	－	Ile80	－	－	*qnr*S, *aac-(6′)-Ib-cr*	UT	D	<1		9
24–1	Dog	6	>256	Leu83	Asn87	Ile80	－	－	*aac-(6′)-Ib-cr*	UT	D	32	ESBL (CTX-M-14)	16
28–1	Cat	6	>256	Leu83	Asn87	Ile80	－	－		UT	B1	<1		12
88–1	Cat	8	>256	Leu83	Asn87	Ile80	－	－		UT	B1	<1		5
107–1	Dog	8	>256	Leu83	Asn87	Ile80	－	－		UT	B1	<1		4
108–1	Dog	8	>256	Leu83	Asn87	Ile80	－	－		UT	B1	<1		4′
57E-1	Cat	12	>256	Leu83	Asn87	Ile80	－	－		UT	B1	<1		11
40–1	Cat	16	>256	Leu83	Asn87	Ile80	－	－		UT	B1	<1		12′
2E-1	Cat	16	>256	Leu83	Asn87	Ile80	－	－		UT	B2	<1		3
73–1	Cat	16	>256	Leu83	Asn87	Ile80	－	－		UT	B2	<1		3′
13–1	Dog	24	>256	Leu83	Asn87	Ile80	－	－	*qnr*B	O1	D	32	AmpC (DHA-1)	1
7–1	Cat	32	>256	Leu83	Tyr87	Ile80	－	Ala458	*aac-(6′)-Ib-cr*	UT	D	16	ESBL (CTX-M-14, TEM-1)	7
42–1	Dog	>32	>256	Leu83	Asn87	Ile80	－	Ala458		UT	D	<1		2
85–1	Cat	>32	>256	Leu83	Tyr87	Ile80	－	Ala458		UT	D	>32	nd ^*r*^	8
76–1	Dog	>32	>256	Leu83	Asn87	Ile80	－	Ala458		UT	B1	<1		14
83–1	Dog	>32	>256	Leu83	Asn87	Ile80	－	Ala458		UT	B1	<1		14
86–1	Dog	>32	>256	Leu83	Asn87	Ile80	－	Ala458		UT	B1	<1		14
96–1	Dog	>32	>256	Leu83	Asn87	Ile80	－	Ala458		UT	B1	<1		14
97–1	Dog	>32	>256	Leu83	Asn87	Ile80	－	Ala458		UT	B1	<1		14
98–1	Dog	>32	>256	Leu83	Asn87	Ile80	－	Ala458		UT	B1	<1		14
105–1	Dog	>32	>256	Leu83	Asn87	Ile80	－	Ala458		UT	B1	<1		14
106–1	Dog	>32	>256	Leu83	Asn87	Ile80	－	Ala458		UT	B1	<1		14
70–1	Dog	>32	>256	Leu83	Asn87	Ile80	－	Ala458		UT	B1	<1		14′
104–1	Cat	>32	>256	Leu83	Asn87	Ile80	Gly84	－	*aac-(6′)-Ib-cr*	UT	D	16	ESBL (CTX-M-14, TEM-1)	15
87–1	Cat	>32	>256	Leu83	Asn87	Ile80	Val84	－	*aac-(6′)-Ib-cr*	O25	B2	>32	ESBL (CTX-M-27)	17

*a,* MICs for ciprofloxacin, nalidixic acid and cefotaxim were confirmed with an E-test.

*b,* Mutation in *gyr*B was not found.

*c,* PMQR: *qnr*A [14], *qnr*B [14], *qnr*C [14], *qnr*D [16], *qnr*S [14], *qep*A [14], *oqx*A [15], *oqx*B [15] and *aac(6')-Ib-cr* [14] were tested.

*d,* Prime symbols (', '', ''', '''' indicate >80 % similarity between band patterns.

*e,* Negative for the shiga toxin gene.

*f,* β-Lactamase phenotype and β-lactamase genes were not detected.

**Fig. 1.**
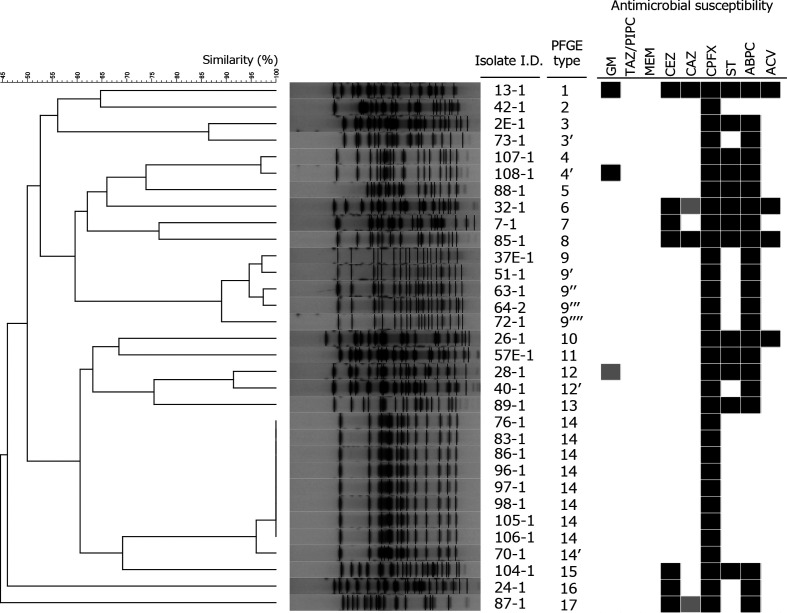
PFGE analysis and antimicrobial susceptibility. *Xba* I-digest PFGE patterns and a dendrogram based on band pattern similarity are shown. For PFGE type, similarity <80 % is indicated as a different number, and ≥80 % is indicated as the same number with prime symbols (′, ′′, ′′′, ′′′′). Susceptibility to gentamicin (GM), piperacillin/tazobactam (TAZ/PIPC), meropenem (MEM), cefazolin (CEZ), ceftazidime (CAZ), ciprofloxacin (CPFX), trimethoprim-sulfamethoxazole (ST), ampicillin (ABPC) and amoxicillin-clavulanic acid (ACV) is indicated by shaded boxes. Black boxes, resistant; grey boxes, intermediate; white boxes, susceptible.

Antimicrobial susceptibility to nine antibiotics was tested (Fig. 1). The proportions that showed resistance or intermediate resistance were as follows: 9.4 % for gentamicin, 0 % for piperacillin/tazobactam, 0 % for meropenem, 21.9 % for cefazolin, 12.5 % for ceftazidime, 100 % for ciprofloxacin, 40.6 % for trimethoprim-sulfamethoxazole, 68.8 % for ampicillin and 12.5 % for amoxicillin-clavulanic acid.

PFGE and a dendrogram analysis were performed to assess genetic relationships (Fig. 1). There were 17 distinct patterns with <80 % similarity from the 32 fluoroquinolone-resistant *E. coli* isolates. The similarity of band patterns ranged from 14.3 to 100 %. There were five PFGE types that indicated >80 % similarity, for a total of 20 isolates (Table 2, Fig. 1).

## Discussion

In this study, 47.4 % of dogs and 17.9 % of cats carried fluoroquinolone-resistant *E. coli *. The reason for the significant difference in prevalence rates between dogs and cats, or between males and females, was not clear; however, isolates showing the same PFGE types were detected in multiple dogs, and this may have influenced the prevalence rates. Additionally, the presence of a previous owner was not necessarily related to possessing the bacteria. Sato *et al*. found that a high prevalence of fluoroquinolone-resistant *E. coli* may have been caused by frequent or continuous fluoroquinolone use in animal hospitals [9]. In the present study, the use of enrofloxacin in the animal shelter was not related to possessing the bacteria, although a full medication history was not determined.

Co-resistance to cephalosporin was observed in 21.9 % (7/32) of fluoroquinolone-resistant isolates. These included four ESBL-producing and two AmpC-producing isolates. Moreover, 68.8 % (22/32) of fluoroquinolone-resistant isolates were also resistant to other categories of antimicrobials. Fluoroquinolone-resistant *E. coli* with co-resistance to other antimicrobials, especially cephalosporin, is prevalent nationwide [3, 4], and has also been detected among clinical *E. coli* isolated from companion animals [10, 17]. According to the international standard definition for multidrug-resistant (MDR) bacteria [18], 15 isolates (46.9 %) were classified as MDR with non-susceptibility to three or more antimicrobial categories. These findings may have great relevance for the treatment of infectious diseases in both animals and humans.

For QRDR, all isolates harboured a mutation in *gyr*A at Ser83, the most common mutation site. Highly resistant isolates carried a combination of mutations within *gyr*A and *par*C, and the number of mutations was proportional to the MIC for ciprofloxacin. In line with previous reports regarding fluoroquinolone-resistant *E. coli* from companion animals [7, 10], mutations in QRDR genes play a significant role in mediating resistance to fluoroquinolone. For PMQR genes on transferable plasmids, which confer a lower level of resistance to fluoroquinolone than QRDR [4, 5], *aac-(6′)-Ib-cr* was the most prevalent, followed by *qnr*S and *qnr*B. The predominance of the *aac-(6′)-Ib-cr* gene and *qnr* variant genes agrees with the previous reports of *E. coli* isolates recovered from companion animals in the USA [10] and Europe [7, 8]. This trend has been confirmed in human clinical isolates as well [3]. Furthermore, many different PMQR genes have also been found in *E. coli* isolates from Chinese companion animals [11].

PFGE patterns were widely diverse, but some isolates showed the same or similar band patterns. Although these animals were from separate locations, cross-contamination may have occurred on admittance to the animal shelter or may also have been caused by the movement of staff or materials.

In conclusion, we found that fluoroquinolone-resistant *E. coli* was widely distributed among sheltered companion animals with a wide range of individual attributes. This may be related to the dissemination of fluoroquinolone resistance among Japanese human clinical *E. coli* [2], livestock animals [19] and different environments [20]. To lower the overall fluoroquinolone resistance, it will be necessary to reduce fluoroquinolone use and continue surveillance not only in the human clinical field but also in the veterinary field.

### Consent for publication

This paper does not include details, images or videos relating to an individual person. Therefore, informed consent is not necessary.

## Data bibliography

This paper does not include data reference.
